# Liebig and Thomson on the Food of Animals

**Published:** 1847-04

**Authors:** 


### ART. XIII.

1. *Animal Chemistry; or Chemistry in its Applications to Physiology and Pathology.* By Baron LIEBIG.—*London,* 1846. 8vo.
2. *Experimental Researches on the Food of Animals and the Fattening of Cattle; with Remarks on the Food of Man.* By ROBERT DUNDAS THOMSON, M.D.—*London,* 1846. 8vo.

THE minute study of the nature and relations of the food of animals and plants has often appeared to us calculated to elucidate, not only the practical points, which are at once obvious even to a superficial observer, but also to throw a light upon several more abstract physiological questions, which have been debated too often with a reference merely to one phase in the history of organized beings. In directing our attention to some of the new views which have been broached upon the nature of food, it may not be out of place briefly to consider the bearing of the subject upon one or two of these disputed points.

A proper distinction between the animal and vegetable world has long been a desideratum with the physiologist. Nor will the circumstance of the question being still undecided appear remarkable, if we reflect that correct notions are scarcely yet formed of the proper constituents of the frames of the two classes of beings, and more particularly that the source of these substances is still disputed. But scientific men have even experienced much difficulty in defining the general characters of vegetables and animals. The former being confined to one spot, and the latter being moveable, some have considered that animals were distinctively locomotive. But it so happens that many of the lower tribes of animals are incapable of locomotion, and hence this definition is untenable. Others, observing that plants are destitute of sensation, have proposed to ascribe this attribute alone to animals, and define them as nervous beings. But in opposition to this view, we find many inferior animals apparently destitute of sensation, and only supplied with a degree of irritability even inferior to that of the sensitive plant, cultivated so frequently in our botanic gardens; and hence this definition also fails us. We believe that the true distinction between animals and vegetables will be detected more readily by discovering the nature of the matter by means of which they increase in bulk, or, in other words, by the nature of their food—the term food being a word applied to express the matter which enables plants and young animals to increase in size, and full-grown animals to preserve their forms unimpaired. According to this view then, it is from chemical physiology that we are to expect an answer to the questions, "What is an animal?" "What is a vegetable?" To one accustomed to view only the larger kinds of animated beings it might seem an easy task to give a reply to these questions. But when we know that nature is simple in her works; that in her we find no sudden leaps from great to small, but that the whole animated world consists of a chain formed of a series of beings passing down in regular gradation from comparatively the most perfect to the most imperfect state, the lowest plant being closely allied to the lowest form of animal, it will at once be obvious that to say where plants begin and animals end cannot be a problem of easy solution.

To apply, however, the test which we have proposed, let us begin with plants. We find a plant cultivated among the Chinese and introduced among ourselves, termed the air-plant, which, by being merely suspended in the air, increases in bulk and weight without even the application of water. This is one of the most simple forms of vegetable life, as the plant has nothing to feed on save the atmosphere, which, however, contains all the elements necessary for its growth—oxygen, vapour of water, carbonic acid, and ammonia or some form of nitrogen. But all these are gaseous bodies or vapours, while the air-plant is a solid; hence we infer that this plant is capable of reducing gases to the solid form, and of thus increasing in bulk and weight. According to the views of persons best qualified to judge, it appears that all plants are endowed with similar properties, and that they mainly subsist by feeding on the gases which surround them, by converting these gases, by means of the organs with which they are supplied, into the solid forms of the vegetable kingdom, so endless in figure, but yet so lovely, that the greatest familiarity only renders them objects of superior admiration.

When we turn to the animal world, we find that the individuals of which it is composed are incapable of condensing gases; in fact the least educated knows that animals derive no sustenance from air; but that they require to imbibe solid matter similar to that of which they consist. Man lives upon animal food, and those kinds of grain which contain matter nearly allied to it. The question, why has grass, perhaps the most abundant vegetable in nature, never constituted a portion of human food, unless perhaps among the most degraded of the species, may not strike us as being in its answer fraught with important information. And yet the only reason which can be given for the fact, that it has never been an article of human food, is, because it contains such a small portion of matter similar in its nature to the constituents of man's frame, that the quantity required would be too voluminous for the digestive capacity of the stomach and other organs. An animal may therefore be defined, according to this view, to be a being which subsists by appropriating to itself food similar to the matter of which its own body is composed. Hence we see the necessity for its locomotion; while a plant, finding its nourishment in the air which constantly surrounds it, has its food brought to it by the usual laws of inanimate nature. We believe, then, that such will be found the most correct mode of separating animals from plants. It is probable that among the inferior tribes of animals, where an approximation is made to the vegetable kingdom, there may be individuals partaking of a semi-vegetable and animal nature, partly living on air and partly on solids. Although it does not follow that such an occurrence is necessary, yet, from the simplicity and gradation which we find subsisting throughout nature, we might expect to discover some such union of the two kingdoms, or some equally simple transition from one set of beings to the other. To discover whether any such series of beings exists, it is obvious that we can proceed upon the principles which we have now been discussing. For example, if we find any usually considered plant or animal possessing in its constitution some substance which we know to be peculiar to a plant, and capable of being produced from gases through the instrumentality of the vegetable organism; and further, if we never find this matter present in animals, we seem to be drawing a legitimate conclusion when we infer that these species partake of a vegetable nature, and that we are approaching a point in creation, where the two great organized kingdoms are closely allied, or insensibly passing into each other.

This investigation has been commenced and has been followed by a successful result in one or two instances. It was observed some years ago by Wöhler, that the *frustulia salina* of Ehrenberg, a small zoophyte found in the slimy matter of saline springs at Königsborn, disengages large quantities of pure oxygen, so that when the mud containing a number of these beings is stirred with a stick, a beer-glass of water inverted may be filled with the gas in a very short space of time. This remarkable phenomenon, which never occurs in the clearly established animal race, but is a characteristic of plants, naturally attracted the attention of chemists, and, accordingly, upon examination by Schmidt, it was found that the constitution of this zoophyte shows that it is closely allied to the vegetable kingdom. A substance was extracted from it, which, after treatment with ether and dilute caustic potash, gave, by analysis, the silica being subtracted, the following composition:—carbon 46·19, hydrogen 6·63. Now these are exactly the numbers which have been obtained as expressing the composition of the basis of that inferior though familiar class of plants the lichens. The inference then seems legitimate, that the substance which constitutes the walls of the cells of at least this class of plants is identical with, in composition with, a substance found in the *frustulia salina*. If we compare the constitution of the cells of decided animals with that of vegetables, we find a sufficient distinction in the presence of nitrogen in the animal, and its absence in the vegetable cell. If we distil each of these matters respectively, we obtain an ammoniacal fluid in the one case, and an acetic fluid in the other,—a distinction pointed out between animal and vegetable substances so long ago as 1742 by Beccaria of Bologna. (Thomson's Researches on Food, p. 158.) All such chemical results, it is obvious, therefore, are important in enabling us to arrive at a conclusion in reference to the nature of the food of different beings, and therefore of the position which these beings hold in the scale of creation. So important, therefore, is an acquaintance with the nature of the food on which such beings subsist even to the physiologist.

Again, in the *cynthia mammillaris*, a species of ascidia, there is a thick fleshy sack connected with the gills, liver, &c., which consists of a congeries of large cells similar to the parenchyma of the cacti and many fruits. Upon its inner side numerous vessels expand, which communicate with the gills. When this outer sack is successively treated with water, alcohol, ether, dilute acids, and alkalies, a colourless membrane remains, which is not altered by nitric, hydrochloric, acetic acids, nor by the most concentrated solution of caustic potash. It is quite destitute of nitrogen, and when analysed, was found to consist of 45·38 per cent. of carbon, and 6·47 of hydrogen, a composition identical with that of the cellular membrane of plants, which has been termed *cellulin* or *cellulose,* and is identical in composition with starch and sugar. In the preparation of the substance as previously described, the solution of potash in a caustic state dissolves up from the cellulin a quantity of azotized matter possessing albuminous properties. So that the cells of the beings examined were quite analogous to those of plants in more than one point of view, as it is an operation of some difficulty to separate the nitrogenous constituents of a plant from the cellular matter in those species on which no doubt exists as to their proper position in the organized scale.

From the observations which have been previously made, it is apparent that before we can point out the proper food destined for animals, we must study carefully their habits and endeavour to discover the food which they prefer when left freely to the enjoyment of their instincts. It is evident that there are certain laws which nature has laid down that serve to guide even the lower animals in the choice of their food. It is a rare occurrence to hear of the suicide or accidental death of a domesticated animal; still less frequent that of a creature which is free to roam amid the wild scenes of nature. We remember one case in which some dried and powdered monkshood (*aconitum napellus*), to the extent of a quarter of an ounce, was licked up by a pony. The animal suffered considerably, as if under an attack of glanders, for a few hours. But these instances are so rare that it might almost be affirmed that man is the only created being which disobeys the laws of nature; and it is merely when domesticated and under circumstances analogous to those in which man himself is placed, that we find the inferior creation imitating by such experiments their more godlike superiors.

It is of essential importance to decide by what method we are to arrive at the nature of the proper food of animals. There must be no *petitio principii.* With all our reverence for Scripture, therefore, we must protest against the conclusions which some well-meaning, but one-sided medical commentators have drawn, as to its being employed as a text-book for all or any of the sciences. Its object was moral, not physical, and the statements that occur in it in reference to science of all kinds, are necessarily such as are consonant with the opinions of those who lived in the earliest ages. If they had been, or could have been otherwise, it is highly probable that these writings would never have reached our times; but have long since been consigned to oblivion as the records of physical impossibilities, and of erroneous speculations in science, or as the dreams of the mystics. Nor are we to expect to derive physical truth from the lucubrations of the often well-meaning, but equally ignorant mere humanity-mongers, who "strain out the gnat and swallow the camel," exhibiting in themselves, as with all ultra-advocates, the most distressing instances of human inconsistency. These are imitators of Pythagoras, not only in their support of a so-called humanity, but also in their advocacy of the principle without consistency. That distinguished philosopher could exclaim against the abominable wickedness that men should permit bowels to be buried in bowels, that one greedy body should grow fat with another body crammed into it, and that one animal should live by the death of another animal, and characterize the use of animal food, as champing with the teeth nothing but dreadful wounds, and thus reviving the manners of the Cyclops. "Why has the sheep deserved death," he asks, with vehemence, "that harmless animal which carries nectar in its full dugs, and furnishes us with soft clothing and aids us more by its life than its death?" At first sight, it is impossible to avoid sympathizing with the energetic and poetical eloquence of such a humane advocate. But the scene is suddenly changed; our sympathies are drawn back to our own bosoms, and another variety of feelings is engendered when we remember that this remarkable philosopher, as if to demonstrate that ultra views necessarily produce inconsistency, gave the lie to all his aspiring sentimentality, by sacrificing in the most cruel manner, and without any ulterior object to serve, 100 oxen in commemoration of his discovery, that the square on the hypothenuse of a right-angled-triangle is equal to the sum of the two squares on the base and perpendicular. So striking is this imperfection in his character, that his apologists suggest, but without any authority, that the oxen *must have been* composed of wax. But let it not be supposed that such opinions have fled before the light of knowledge, which has been promulgated during the 2344 years that have intervened since his death. For we find the same school of humanity-mongers, at the termination of that long period of time, still inculcating the idea in reference to the use of animal food, that "the man of cultivated moral feelings shrinks from the task of taking the life of the higher grade of animals, and abhors the thought of inflicting pain and shedding blood," just as if this were a correct statement of the question. While we therefore protest against every needless occasion of suffering, to all classes of animated nature, we should characterize such bastard humanity as we have alluded to as beneath consideration, nay in many cases as insufferable, if carried out with any semblance of consistency.

Some of the exclusively vegetable dietists, who base their doctrines upon a kind of one-sided metaphysico-historical research, commence by asking and solving the question as to the original food of man, not by the simple physiological method of observing the nature of the food with which the mother is supplied by nature for the support of the *original* man, but by speculating respecting the food used in the garden of Eden. The primary and original food of man, whatever speculators may say to the contrary, is milk, a fluid of animal origin. If those who are to regulate diet are not guided by scientific knowledge, and are not to exercise their judgment, they might be inclined to draw from this fact the conclusion that the proper nutriment of man is animal food, and this deduction might be defended with great show of reason to the exclusion of a vegetable diet altogether. But observation having proved that animals can subsist upon a vegetable as well as upon an animal regimen, and scientific research having satisfactorily demonstrated that the constituents of the two kinds of nutriment when well selected are identical, the one-sided position which might have previously been assumed and strenuously maintained, must yield to the lights of knowledge.

A careful examination of the constituents of the milk supplied by nature to animals, proves to us, as originally suggested by Dr. Prout, that a nitrogenous substance is required in our food, together with a representative of the oily and saccharine classes of substances. To Liebig we are indebted for the view which enables us to discriminate between the relative values of these species of nutriment; the nitrogenous food according to him, serving to supply the waste of the muscular tissues, while the unazotized materials act as agents in the process of respiration. An additional importance has lately been attributed not only to the necessary existence of such classes of substances in the food, but likewise to the proportion in which they exist in the different kinds of nourishment used by animals under different circumstances. (R. D. Thomson on the Food of Animals. &c. p. 167.) For example, we find the composition of the milk of women, the ass, and the cow, to be in reference to the amount of curd, sugar, and butter, nearly as follows.

| | Woman. | Ass. | Cow. |
|---|---|---|---|
| Casein . . . . | 1·52 | 1·95 | 4·16 |
| Butter . . . . | 3·55 | 1·29 | 3·70 |
| Sugar . . . . | 6·50 | 6·29 | 4·35 |

If we divide the amount of butter and sugar by the amount of casein, we shall obtain the relation of the nitrogenous to the non-nitrogenous principles. The relations are as follows:

| | |
|---|---|
| In woman's milk the relation of casein to sugar and butter is as . . | 1 to $6\frac{1}{2}$ |
| In asses' milk " " " . . | 1 to $3\frac{3}{4}$ |
| In cow's milk " " " . . | 1 to 2 |

We observe, therefore, that in the food of growing animals, there is a difference in some degree in the relation between that portion required to supply the waste of the system, and that employed in respiration or rather for the production of animal heat, depending undoubtedly on the nature of the animals. In practice we act upon this principle without perhaps appreciating its force; for when we are required to substitute cow's milk for the support of the human infant instead of its mother's milk, we add sugar and dilute it with water. Both of these precautions are judicious, but the former, in accordance with the view now taken of the subject, is particularly advisable. In confirmation of the existence of a relation between the two series of constituents of the food, it deserves attention, that all the analyses of the different kinds of milk which have hitherto been made prove that their composition is steady, and undergoes comparatively little variation. It is pretty obvious that from this circumstance alone, even if it were not strengthened by an abundant induction from other facts, that we may derive important knowledge with regard to the nature of human food. For when the milk is converted by the stomach into a proper condition for being absorbed, and is presented to the open mouths, it may be of the lacteals in its descent through the intestinal canal, we cannot understand how its constituents should be taken up by these absorbents in a proportion different from that which prevails in the natural condition of the fluid. Indeed we have evidence that in the healthy condition of the digestive organs of an infant, almost the whole of the constituents of the milk are absorbed, from the fact that when the fæces of a sucking child are examined under the microscope, they are seen to consist mostly of epithelial cells derived from the mucous membrane of the intestines (Liebig.) If this be the fact with regard to milk, we may expect that it will also hold in reference to any other kind of aliment which the stomach is capable of digesting, that is of preparing it in a condition proper for being absorbed by the lacteals. And this conclusion we are compelled to adopt, unless we were to have recourse to the ancient exploded doctrine that the absorbents are endowed with a discriminating power which enables them to select what is proper for making good blood, and rejecting what would be injurious. If this doctrine were true, then poisons when swallowed internally ought to be innoxious, and the causes of diseases operating from within should uniformly be effete. But even the analogy of the vegetable kingdom exhibits the fallacy of such a view, and the beautiful process of variegating wood by causing chemical solutions of various kinds to be absorbed from the roots, and being conveyed by the sap to permeate the hardest portion of the monarch of the woods, is a sufficient answer to such notions. These considerations tend to show that not only must the food contain certain ingredients; but that these proximate elements must be present in the proper proportion.

The contrast of another species of food often administered to children with milk, which all must agree to be the type of the natural food of the infant man, will enable us to form an idea of the importance of the relationship which has now been insisted on. Arrow-root, for example, is almost universally given as infant food, and it belongs to the class of what is designated farinaceous diet. The following is the approximate composition of some of these substances per cent.:

| | Arrow-root. | Tapioca. | Sago. |
|---|---|---|---|
| Starch, &c. . . . | 84·29 | 83·87 | 84·87 |
| Albuminous matter . . | 3·21 | 3·13 | 3·33 |
| Water . . . | 12·50 | 13·00 | 11·80 |

In these articles of diet, the starch occupies the position which the sugar and oil represent in the milk. Hence if we divide the starch by the albuminous matter, we obtain the relation between the nitrogenous matter and heat-producing substance (calorifiant matter of Dr. Thomson) of 1 to 25. If we consider, for the sake of conciseness, these numbers to represent particles of the constituents of the food, we shall find that when a child consumes milk and arrow-root at separate meals, the nature of its fluids must be completely altered at these several times, since in the case of milk we have presented to the mouths of the lacteals in a digested state, 1 particle of nitrogenous matter and 6 particles of calorifiant matter, while with arrow-root 1 part of muscular substance and 25 of heat-producing materials are exhibited to the extremities of the absorbents, and carried into the circulation so far as we are capable of judging from an examination of the excretions. Under such circumstances, it is obviously impossible that the blood can possess a steady composition. The great predominance of calorifiant matter must give rise to the formation of substances which cannot be removed from the system with sufficient rapidity; while the muscular system cannot be sufficiently sustained in consequence of the necessary nutriment being withheld, and its place supplied with matter which ought ultimately to be excreted in a volatile condition. It is true that the arrow-root class of food is usually administered along with milk; but although this may afford some palliation for the mistake of considering arrow-root as a sufficient substitute for milk, it affords after all but a slight amelioration of the fallacy. For if we represent the arrow-root and milk by a formula, by substituting such a diet for milk alone, we should commit the error of representing milk + arrow-root, as equal to milk + milk. And thus we should be landed in the exact position from which we had endeavoured to escape by the preceding subterfuge; that is, if we persisted in recommending arrow-root as a fit diet for children, we should be compelled to prove that it possessed the same composition as milk, or that its proximate constituents were capable of being transformed into the same substances and in the same proportions as exist in milk.

It does not follow that an error of this nature should be followed by any immediate result of such a description as to excite alarm, but it may act as a prelude to the access of dangerous causes of disease, or in the common language of medical writers, it may afford a predisposing cause for disease. A remarkable confirmation of the view which is here given, was afforded by an experiment made by Mr. Smith of Deanston, the distinguished agriculturalist. At a time when sago was universally recommended as a cheap material for feeding cattle, he purchased a quantity of it, and employed it for fattening a number of calves, substituting the sago for a certain amount of milk. The animals appeared to thrive and grow fat, but in the course of the following year every animal so fed died, some from inflammation, and others from incidental diseases, the accession of which he attributed to the use of the sago, the calves fed on milk alone, exhibiting no symptoms of unusual unhealthiness. This statement was made at a public meeting by Mr. Smith himself, after the previous view of the proper constitution of food had been explained, and is therefore an application due, not to the author of the view, but to one of extensive practical experience, who obtained the result without any reference at the time to the essential nature of the cause. This view, then, is directly opposed to the doctrine of Boussingault, to which reference was made in a former Number of this Review. That chemist states that the equivalent amount of different kinds of food may be determined by ascertaining the quantity of nitrogen which each contains. Every substance by this view, which therefore is possessed of nitrogen, is equally as good as any other, if the equivalent be used. Thus, 100lbs. of hay are equal to 612lbs. of turnips, or 400 of beetroot, or 281 of potatoes in reference to the feeding of cattle. But such an idea is at once refuted by the most common experience. Because, according to the same principle, 281lbs. of potatoes should answer as well for feeding horses as 54lbs. of oats, But every one knows that the greatest possible quantity of potatoes could never replace any quantity of oats in the nourishment of horses. The view which has been previously given affords a satisfactory reason why no such substitution is adequate. The following table supplies us with a view of the relation between the constituents of different articles of food, (R. D. Thomson, on the Food of Animals, p. 167) :—

| | Relation of nutritive to calorifiant food. |
|---|---|
| Milk—food for a growing animal | 1 to 2<br>1 to 6½ |
| Beans | 1 to 2½ |
| Oatmeal | 1 to 5 |
| Semolina<br>Barley | 1 to 7 |
| Wheat flower—food for an animal at rest | 1 to 8 |
| Potatoes | 1 to 9 |
| Rice | 1 to 10 |
| Turnips | 1 to 11 |
| Arrow-root, Tapioca, Sago | 1 to 26 |
| Starch | 1 to 40 |

This table (which, however, can only be considered as approximate, since the proportion between the proximate constituents of food is constantly undergoing slight modifications) enables us to perceive that we can never expect such a substance as potatoes to replace such species of food as beans or oats without producing a decided prejudicial influence upon the animal so treated, more especially if the animal were exposed to any degree of muscular exertion. Suppose, for example, we represent the amount of muscle removed from the body of a horse to be 2lbs. per day, while the amount of food consumed in the production of heat was 12lbs., it is at once obvious that, to make up for this loss, we should never think of giving to the animal, food containing 2lbs. of albuminous or muscular matter and 52lbs. of calorifiant matter, that is, sago; neither should we give a diet containing 2lbs. of albumen and 22lbs. of calorifiant matter, that is, turnips. But we should endeavour to administer nourishment which contained as nearly as possible the ingredients which the animal's consumption required. This object would be nearly attained by the use of oats, which would give, for every 2lbs. of albumen, 10lbs. of calorifiant matter, or by barley, 2 to 14. A mixture, then, of the two grains would supply the deficiency required by the animal; or the same result would follow by the employment of beans and hay. In short, we can understand at once from this insight which we attain of the composition of food the whole rationale of the usual practical mode of feeding animals of all descriptions. "The animal system is thus in an analogous condition to a field from which different crops extract different quantities of matter from the soil, which must be determined by experiment." (On the Food of Animals, p. 168.)

We have already seen how nature provides for the different circumstances in which the varied species of animals are placed, by modifying in some degree the constitution of the milk. Judging from this fluid, we should be led to infer that the calf and young ass undergo more muscular exertion than the human infant, without any acquaintance with the habits of the animals; and that the calf wastes more muscular matter than the ass.

There are some animals which apparently make no use of calorifiant matter. How, then, it may be inquired, is their animal heat kept up? The American savage, for example, consumes animal food alone, and beasts of prey are nourished after a similar fashion, without having an opportunity of partaking of the fruits of the field; for, as they do not sow, it is impossible for them to reap. Yet their animal heat is sustained; not, however, it must be observed, without great exertion, and a restless and wandering existence impressed on them in a great degree by the nature of the food upon which they subsist. All the food which they eat, with the exception of fat, which is, however, always present to a certain extent in muscular tissue (Liebig), enters into the solid constitution of their frames; and it is only when the muscular substance of the body is passing into gelatinous and other soluble states that it is capable of evolving heat. This degradation of muscular tissue must proceed even in an animal in a perfect state of rest; but it is accelerated by motion in proportion to the degree and violence of the exercise. To keep up the animal heat, more particularly in winter, when it is tending to radiate and to be conducted away by the colder surrounding media, the savage is compelled to cover himself with warm skins and to take a great amount of exercise. These expedients serve likewise as antagonists to the action of the atmosphere, and assist in forming and retaining heat, which is but tardily extricated. The function, then, which the calorifiant food fulfils in the animal economy, is at once obvious from this view. It is to save the waste of the muscular tissue and to admit of the formation of a sufficient amount of heat by a direct process of combustion. It is not a little remarkable that this physiological condition should constitute an essential element in civilization, by enabling the body to fulfil its functions without violent exertion, and to admit of unrestrained exercise of the mental powers.

There is an important subject of inquiry, upon which much light requires to be thrown before precise notions can be entertained of the true office of the constituents of the food. There seems little doubt that the fibrinous portion of the food is either freely divided or dissolved by the digestive action of the stomach, and is in this state absorbed by the lacteals. But what becomes of the starch is a question which has occasioned much labour of late among chemists. If we feed a hungry pig upon porridge, and in three hours, having killed the animal, filter the contents of its stomach, we find that the filtered liquid does not afford a blue precipitate with tincture of iodine, which it would do if a solution of starch were present; but we obtain a purple colour, indicative of the presence of dextrin or soluble starch. In addition to this fact, we observe that the fluid has a strong acid reaction, resembling that which can be detected in the process of starch making, when wheat flour is allowed to stand in contact with water. This acid possesses the properties of lactic acid. (Thomson on Food of Animals, p. 20; and Philosophical Mag., April and May, 1845.) Besides the production of the dextrin and lactic acid at the expense of the starch, we can demonstrate the presence of sugar in the stomach, having all the characters of that procurable by artificial means from starch. It is obvious that the lactic acid is not absorbed in its free state by the lacteals, because the fluids contained in these vessels are alkaline. There is no reason to suppose that dextrin enters these vessels. The most probable idea is, that the greater portion of the starch is converted into sugar, and that the acid is neutralized by the soda of the bile. That sugar enters by the lacteals appears highly probable from the experiments of Prout, who found a trace in the chyle of a dog fed on vegetable food; and a similar result has been obtained with reference to a trial for sugar in the blood by Dr. R. D. Thomson. (Phil. Mag., 1845, ut supra.) Still more lately Magendie states that he found sugar in the blood of a dog fed on potatoes (Journ.

de Pharmacie, Janvier, 1847), and also a substance resembling dextrin. It must also be stated, however, that Liebig endeavoured to detect sugar in the blood unsuccessfully. In such experiments, much must depend upon the method of investigation, and, as all those hitherto employed are liable to objection, it does not appear that we can give a distinct explanation of the destination of the sugar after it has left the intestines, although there is considerable evidence and probability in favour of the idea that a portion of the sugar derived from the starch of the food may circulate to a certain extent in the sanguineous system. But the circumstance which we observe, even out of the body, that starch and sugar rapidly change their forms when in contact with moist albuminous matter, would negative the notion that any considerable amount of sugar can exist in the normal blood. The rapidity, too, with which sugar can be converted into carbonic acid, renders the prospect still less likely. When we compare the formulæ of sugar and carbonic acid as represented by Liebig, we see that the only difference between these bodies is the replacement of hydrogen in the sugar by the oxygen of the atmosphere. (Liebig's Animal Chemistry, 3d edition, p. 108.)

| Twelve atoms carbonic acid. | One atom grape sugar. |
|---|---|
| $C_{12} \begin{matrix} O_{12} \\ O_{12} \end{matrix}$ | $C_{12} \begin{matrix} H_{12} \\ O_{12} \end{matrix}$ |

It is not necessary that the volatile form should be assumed at once by the sugar. On the contrary, it is probable that various intermediate stages are produced, such as, first, alcohol; then acetic acid, or lactic, butyric acids, &c., all tending to ultimate termination in carbonic acid, as represented by Liebig's formulæ.

| Alcohol. | Acetic acid. | Formic acid. | Carbonic acid. |
|---|---|---|---|
| $C_8 \begin{matrix} H_{12} \\ O_4 \end{matrix}$ | $C_8 \begin{matrix} H_8 \\ O_8 \end{matrix}$ | $C_8 \begin{matrix} H_4 \\ O_{12} \end{matrix}$ | $C_8 \begin{matrix} O_4 \\ O_{12} \end{matrix}$ |

But when such formulæ are exhibited to represent our notions respecting our present views of any chemico-physiological process, it should be borne in mind that the authors of such formulæ merely give them as indicating the direction in which such changes may occur, not as being absolute expressions of fact; and this must be considered as a great step in the progress of physiology. Within but a few years we had not the most distant idea that starch could be converted into the principal constituent of butter; but we now understand how this can be effected, and thus are able to appreciate the connexion between farinaceous and oily food, two classes of bodies which were formerly placed at the greatest possible distance from each other in our chemical arrangements, but which can now be made to pass rapidly into each other. Such inquiries as that to which we have alluded, together with many others which have of late occupied the attention of chemists, have originated in theoretical views, which, although not accurate in all their details, have been proved by subsequent researches to have been made in the right direction. Numerous voyages have been made towards the Arctic and Antarctic Poles: those who have commanded them, it is true, have not succeeded hitherto in reaching the desired goal; but they have all conducted their expeditions with proper precautions, and each voyager has added to the knowledge acquired by his predecessor.

